# Overexpression of FNTB and the activation of Ras induce hypertrophy and promote apoptosis and autophagic cell death in cardiomyocytes

**DOI:** 10.1111/jcmm.15533

**Published:** 2020-06-24

**Authors:** Jie Ding, Yu X. Chen, Yan Chen, Yun Mou, Xiao T. Sun, Dong P. Dai, Chen Z. Zhao, Jian Yang, Shen J. Hu, Xiaogang Guo

**Affiliations:** ^1^ Institute of Cardiology The First Affiliated Hospital College of Medicine Zhejiang University Hangzhou China; ^2^ Echocardiography and Vascular Ultrasound Center The First Affiliated Hospital College of Medicine Zhejiang University Hangzhou China

**Keywords:** apoptosis, autophagy, Farnesyltransferase, hypertrophy, MAPK pathway, rat neonatal ventricular cardiomyocytes, recombinant adenovirus

## Abstract

Farnesyltransferase (FTase) is an important enzyme that catalyses the modification of protein isoprene downstream of the mevalonate pathway. Previous studies have shown that the tissue of the heart in the suprarenal abdominal aortic coarctation (AAC) group showed overexpression of FTaseβ (FNTB) and the activation of the downstream protein Ras was enhanced. FTase inhibitor (FTI) can alleviate myocardial fibrosis and partly improve cardiac remodelling in spontaneously hypertensive rats. However, the exact role and mechanism of FTase in myocardial hypertrophy and remodelling are not fully understood. Here, we used recombinant adenovirus to transfect neonatal rat ventricular cardiomyocytes to study the effect of FNTB overexpression on myocardial remodelling and explore potential mechanisms. The results showed that overexpression of FNTB induces neonatal rat ventricular myocyte hypertrophy and reduces the survival rate of cardiomyocytes. FNTB overexpression induced a decrease in mitochondrial membrane potential and increased apoptosis in cardiomyocytes. FNTB overexpression also promotes autophagosome formation and the accumulation of autophagy substrate protein, LC3II. Transmission electron microscopy (TEM) and mCherry‐GFP tandem fluorescent‐tagged LC3 (tfLC3) showed that FNTB overexpression can activate autophagy flux by enhancing autophagosome conversion to autophagolysosome. Overactivated autophagy flux can be blocked by bafilomycin A1. In addition, salirasib (a Ras farnesylcysteine mimetic) can alleviate the hypertrophic phenotype of cardiomyocytes and inhibit the up‐regulation of apoptosis and autophagy flux induced by FNTB overexpression. These results suggest that FTase may have a potential role in future treatment strategies to limit the adverse consequences of cardiac hypertrophy, cardiac dysfunction and heart failure.

## 1 INTRODUCTION

Heart failure is the most common consequence of several forms of cardiovascular diseases and one of the leading pathological causes of mortality worldwide.^1^ Remodelling of the left ventricle in response to pathophysiological stimuli is an active process that contributes to the development and progression of heart failure. In general, cardiac hypertrophy is considered as an initially adaptive response to chronic pressure or volume overload through the normalization of wall stress and ejection function.^2^ However, prolonged and excessive pathological hypertrophy may lead to a maladaptive state of contractile dysfunction and cardiac decompensation and even serve as an independent risk factor for death.^3^, ^4^ In the process of ventricular remodelling, the changes of cardiomyocytes also involve the loss of the number of cardiomyocytes owing to apoptosis, necrosis, fibroblast proliferation and fibrosis.^5^, ^6^, ^7^

Apoptosis is an evolutionarily conserved and inducible cell death programme that is extremely rare in normal terminally differentiated cardiomyocytes.^8^ However, any substantial increase in the apoptosis of cardiomyocytes may play a significant pathophysiological role in the progression from ‘compensated’ myocardial hypertrophy to ‘decompensated’ heart failure.^9^, ^10^ In contrast to apoptosis, autophagy is initially described as a cell‐protective survival mechanism in heart diseases. Studies have suggested that autophagy in cardiomyocytes is an essential component of the normal process underlying the maintenance of cellular homeostasis and protects the cardiac functions during heart failure.^11^, ^12^ However, studies have demonstrated cardiomyocyte death triggered upon excessively provoked autophagy, which may contribute to the pathogenesis of heart failure.^13^, ^14^, ^15^ Apoptosis and autophagy constitute the two self‐destructive processes that may be triggered and modulated by common upstream signals, although the mechanisms of autophagy and apoptosis are different.^16^ Crosstalks and interactions between autophagy and apoptosis have been confirmed and thought to be very complex and important in the development of cardiovascular diseases.^17^, ^18^

Farnesyltransferase (FTase), an important branching enzyme downstream of the mevalonate (MVA) pathway, catalyses the farnesylation modification that resulted in attaching farnesylated proteins to the inner surface of the plasma membrane and mediated the activation of the downstream small GTPases, particularly Ras protein.^19^ Inhibition of FTase by inhibitors (FTI) was found to attenuate cardiomyocyte hypertrophy and cardiac remodelling and prevent both the onset and late progression of cardiovascular diseases.^20^, ^21^ Previous studies of our team have also shown that the FTase expression and Ras activation were significantly up‐regulated in the pressure overload‐induced chronic cardiac remodelling mouse/rat model and FTI‐276 could attenuate cardiac remodelling in spontaneously hypertensive rats.^22^, ^23^, ^24^ However, the precise role and mechanisms of FTase underlying cardiomyocyte hypertrophy and myocardial remodelling are unclear. FTase shares a common α‐subunit with the protein geranylgeranyltransferase (GGTase) but has unique β‐subunits that dictate substrate specificities.^25^ In the present study, FTase beta (FNTB) was overexpressed in cultured rat neonatal cardiomyocytes with a recombinant adenovirus to clarify its potential role in myocardial remodelling.

## 2 MATERIALS AND METHODS

### 2.1 Isolation and culture of rat neonatal cardiomyocytes

Animal experiments were performed as per the Guide for the Care and Use of Laboratory Animals (NIH Publication, 8th Edition, 2011) and approved by the Institutional Animal Care and Use Committee of Zhejiang University (Zhejiang, China).

Neonatal Sprague Dawley rats were purchased from the SLAC Laboratory Animal Co., Shanghai, China. Pups aged between 24 and 48 hours were used. Primary ventricular cardiomyocytes were isolated and cultured according to previous publications^26^, ^27^ (Supplementary Material).

### 2.2 Generation of recombinant adenovirus

Recombinant adenovirus vectors carrying rat FNTB (AdFNTB) and GFP (AdGFP) were constructed using pAdMax™ vector system (Microbix, Canada) as previously described.^28^ The recombinant adenoviruses only containing GFP (AdGFP) were used as a negative control. (see Supplementary Material).

### 2.3 Immunofluorescence staining

For immunofluorescence experiments, NRCs were cultured on coverslips coated with collagen type I (rat tail collagen, BD) and then fixed with 4% paraformaldehyde (Solarbio, Beijing, China) for 25 minutes. The specific methods of immunofluorescence can be found in the supplementary materials. Coverslips were mounted with a mounting medium fortified with 4′,6‐diamidino‐2‐phenylindole dihydrochloride (DAPI, Abcam) and images were acquired with confocal scanning microscopy (Nikon A1R, Japan), followed by analysis with NIS‐Elements Viewer software (version 4.20, Nikon, Japan). Quantitative analysis of images was carried out using ImageJ analysis software.

### 2.4 Cell viability assay

NRCs were seeded in 96‐well plates at 1 × 10^4^ cells/well in 100 μL culture medium. After treatment, cell viability was evaluated with WST‐8 cell counting kit (Cell Counting Kit [CCK]‐8; Dojindo, Kumamoto, Japan) according to the manufacturer's instruction. Briefly, 10 μL of CCK‐8 solution (1/10 dilution) was added to each well at the end of the experiment and the cells were incubated for 2 hours at 37°C. Absorbance at 450 nm wavelength was measured to determine the optical density value with a microplate reader (Thermo Fisher Scientific).

### 2.5 Detection of the mitochondrial membrane potential ΔΨm by JC‐1

JC‐1 is known to be predominately localized in mitochondria and represents a reliable fluorescent probe for the assessment of their mitochondrial membrane potential ΔΨm. Mitochondrial membrane potential was evaluated by JC‐1, according to the protocol of detection kit.

### 2.6 Terminal deoxynucleotidyl transferase‐mediated dUTP nick end labelling (TUNEL) assay

The TUNEL assay was performed using the TUNEL Apoptosis Assay Kit (BBI, Sangon Biotech, Shanghai, China) according to the manufacturer's protocols. The fluorescence intensity was monitored under a confocal scanning microscope (Nikon A1R, Japan).

### 2.7 Flow cytometric analysis of apoptosis

To quantify apoptosis rate, flow cytometry assay was conducted with phycoerythrin‐conjugated annexin 5 (ANAX5‐PE) and 7‐aminoactinomycin D (7‐AAD) double staining kit (PE Annexin V Apoptosis Detection Kit I, BD) according to the manufacturer's instructions. The cells were resuspended in a binding buffer at a density of 1 × 10^6^ cells/mL after being rinsed twice with ice‐cold PBS. Annexin V‐PE and 7‐AAD were added, and the cells were incubated for 15 minutess in the dark at room temperature. After incubation, the cells were analysed with a flow cytometry (BD Fortessa) within 1 hour.

### 2.8 Transmission electron microscopy (TEM)

For TEM analysis, NRCs were fixed with 2.5% glutaraldehyde at 4°C for 4 hours and post‐fixed with 1% osmium tetroxide for 1 hour at room temperature. The cells were negatively stained with 2% uranium acetate for 30 minutes and subsequently processed through gradient dehydration with graded alcohols, embedding and polymerization. Ultra‐thin sections (60‐80 nm) were obtained using a ultramicrotome and mounted on 300‐mesh copper grids. The sections were stained with uranyl acetate and lead citrate. To identify autophagy and intracellular autophagosomes, the sections were examined under a transmission electron microscope (Tecnai G2 Spirit 120 kV, Thermo FEI, Czech).

### 2.9 Small GTPase activation assay

Ras activation was quantified with the Ras activation G‐LISA assay kit (Cytoskeleton).

In general, small GTPase activation is very transient and immediately reduces to basal levels. For Ras activation assay, G‐LISA experiment conditions were established according to the technical guides of the manufacturer and previous publications.^29^, ^30^ After 24 hours from transfection with AdFNTB or AdGFP, the cells were cultured in a serum‐free medium for 2 hours and treated with DMSO or Ras inhibitor salirasib (25 μmol/L) for 20 minutes. Preparation of cell lysates and Ras activation assay was performed as described in the protocol of G‐LISA kit.

### 2.10 Western blot analysis

After treatment, total proteins were immediately extracted from NRCs using a cell lysis buffer (Cytoskeleton) containing protease and phosphatase inhibitor cocktail (Roche). The lysate protein concentrations were measured with the BCA Protein Assay Kit (Thermo Fisher) and equalized using ice‐cold lysis buffer. Western blotting and immunoprecipitation were performed as described in Supplementary material. Images were captured using Chemi XR5 detection system (Syngene) and analysed with ImageJ analysis software.

### 2.11 Quantitative reverse transcription‐polymerase chain reaction

Real‐time polymerase chain reaction (RT‐PCR) was carried out to detect mRNA expression. Total RNA was extracted from NRCs using TRIzol reagent (Invitrogen) according to manufacturer's instructions. The concentration of RNA was quantified with a NanoDrop spectrophotometer (Thermo Fisher), and the RNA was transcribed into cDNA using a PrimeScript cDNA Synthesis Kit (Takara, Japan). For real‐time quantitative PCR, SYBR Green Premix Ex Taq II (Takara, Japan) was used on 480 II PCR Sequence Detection System (Roche). Relative mRNA expression level was normalized to the expression level of *GAPDH* and expressed relative to that of the control group. Primer sequences used are listed in Table 1 (Supplementary material).

### 2.12 Monitoring autophagic flux by the tandem fluorescent‐tagged LC3 (mCherry‐GFP‐LC3B)

To monitor autophagic flux, cardiomyocytes were transfected with a tandem fluorescent mCherry‐GFP*‐*tagged LC3 construct as previously reported.^31^ The expression of GFP and mCherry was visualized with the confocal scanning microscope (Nikon A1R, Japan). Both GFP and mCherry can be detected in early autophagosomes as yellow puncta. Once the autophagosome and lysosome fuse to form the late autophagolysosome, the green fluorescence quenches and disappears due to the degradation of GFP by the acid lysosomal protease, and the autophagolysosomes were detected as red puncta. Autophagic flux was evaluated by the colour change of GFP/mCherry. Bafilomycin A1 is a vacuolar ATPase inhibitor, an inhibitor of autophagy flux, which prevents the fusion of autophagosomes and lysosomes to form autophagolysosomes. Images were captured at 8h after addition of bafilomycin A1 (100 nmol/L) or DMSO.

### 2.13 Statistical analysis

Data were presented as mean ± standard error of mean and analysed using GraphPad Prism 6.0 software. Statistical significance was evaluated using one‐way analysis of variance (ANOVA) followed Bonferroni/Dunn post hoc test as appropriate. Comparisons between two groups were assessed with an unpaired two‐tailed Student's t test. A value of *P* < 0.05 was considered statistically significant.

## 3 RESULTS

### 3.1 Adenovirus‐mediated overexpression of FNTB in rat neonatal cardiomyocytes

**FIGURE 1:**
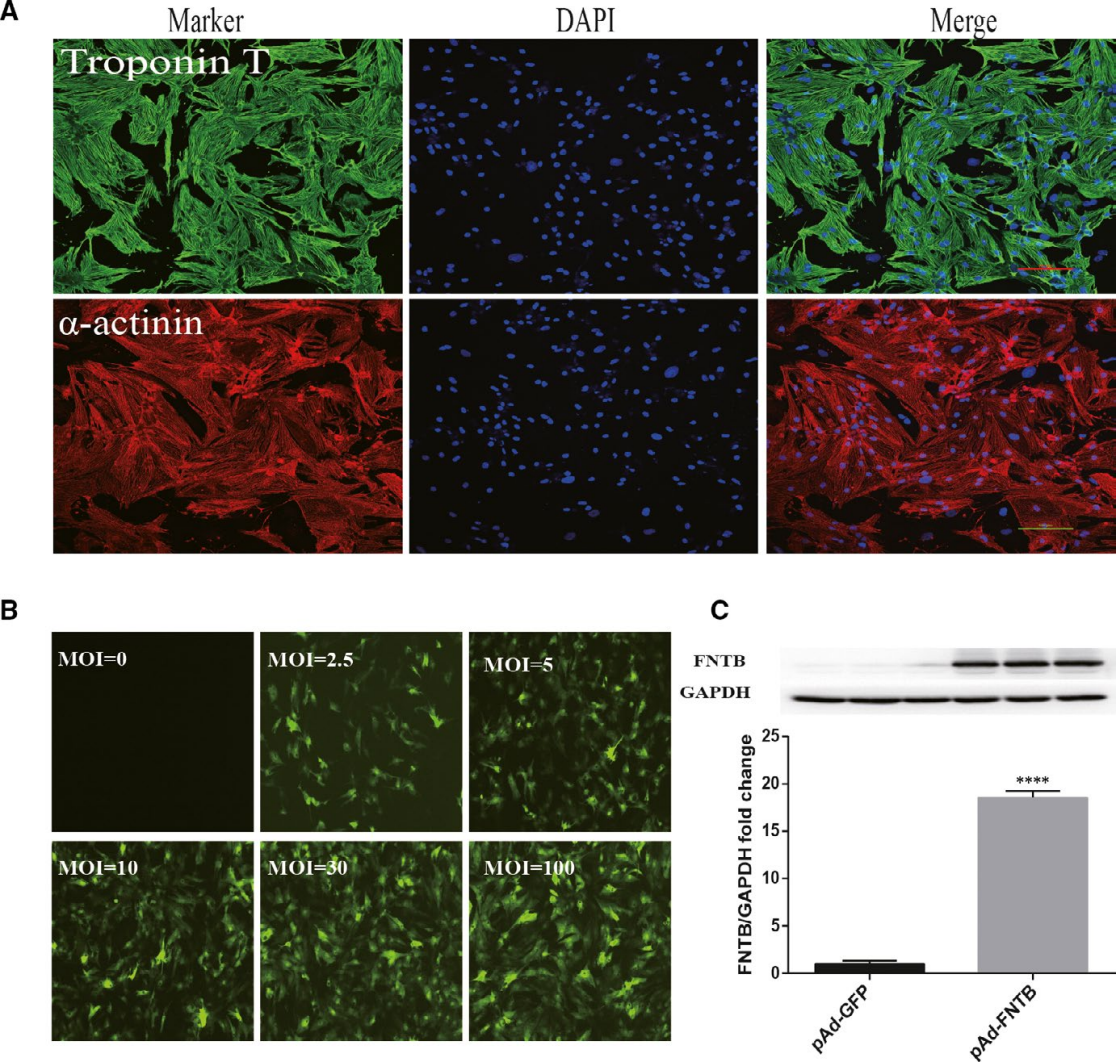
Adenovirus‐mediated overexpression of FNTB in rat neonatal cardiomyocytes. A, Rat neonatal cardiomyocytes were stained with the specific marker α‐actinin (red) and troponin T (green), and cell nucleus was stained with DAPI (blue). Scale bar: 100 μm. B, Cardiomyocytes were infected with AdGFP at various MOIs, and GFP expression was detected by fluorescence microscopy (100×) after 48‐h infection. C, The protein expression of FNTB was tested by Western blot analysis. Data were normalized to GAPDH level and expressed as a fold increase over levels for AdGFP control. ****P < 0.0001 versus control

To confirm the presence and purity of cardiomyocytes, the cultured cells were stained with the specific marker α‐actinin and troponin T, respectively (Figure 1A). We infected cardiomyocytes with adenovirus vectors at MOIs ranging from 2.5 to 100 and examined the expression of GFP after 2 days with fluorescence microscopy (Figure 1B). According to GFP expression observed with fluorescence microscopy after 48 h of adenoviral infection, multiplicity of infection (MOI) of 30 was finally selected as the optimum dose for NRC transfection in the following experiments. Western blot analysis was conducted to relatively quantify the effects of FNTB overexpression (Figure 1C).

### 3.2 Activation of Ras is up‐regulated in rat neonatal cardiomyocytes upon FNTB overexpression

**FIGURE 2:**
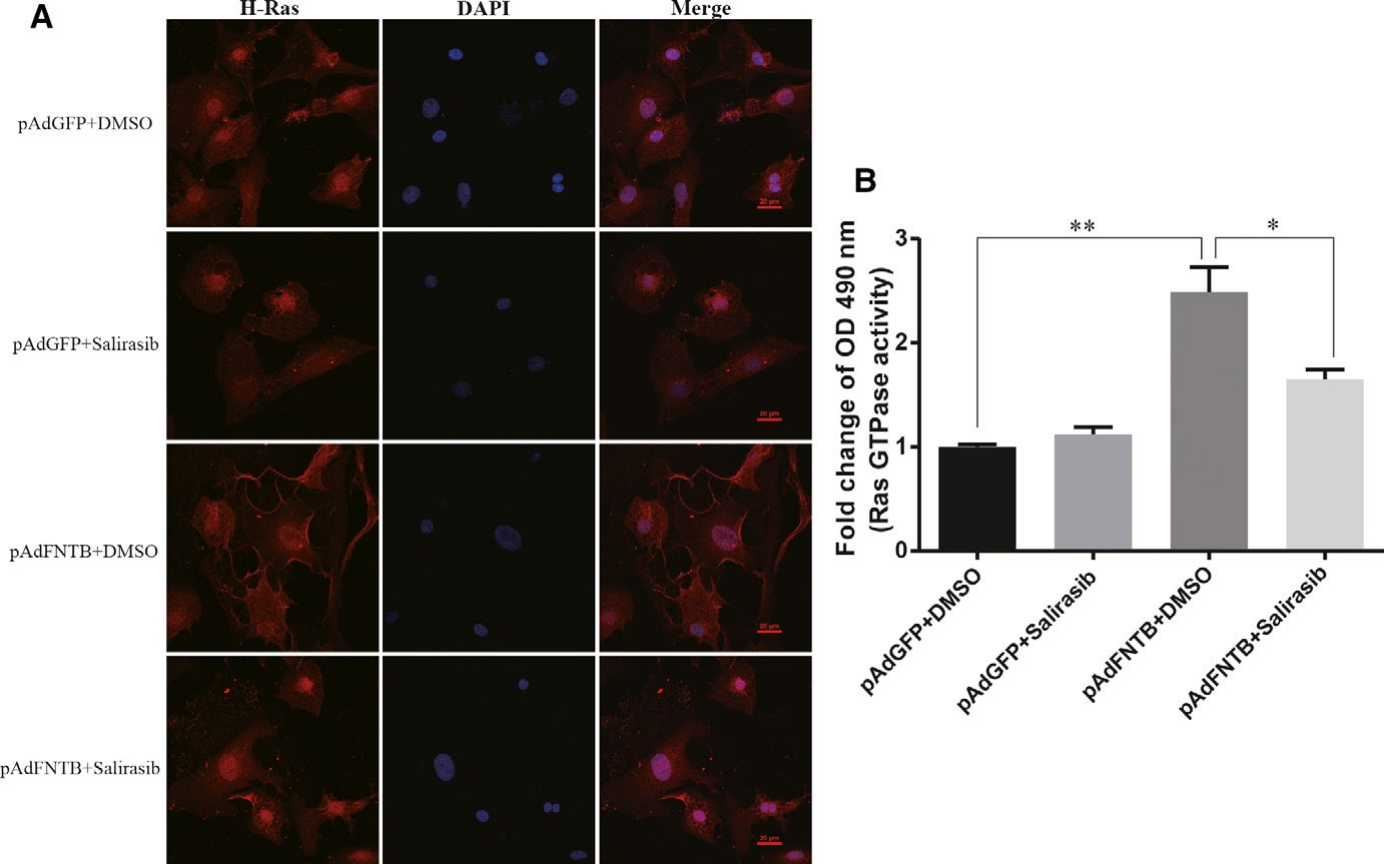
FNTB overexpression mediates Ras activation. A, The cellular localization of H‐Ras in cultured neonatal cardiomyocytes was examined using confocal microscopy. Ras (red) expression was detected with anti‐H‐Ras antibody and Alexa Fluor 594‐conjugated secondary antibody. DAPI (blue) was used as a nuclear counterstain. Scale bar: 20 μm. B, Ras activation was quantified with the G‐LISA assay. Mean ± SEM from 3 independent experiments, **P < 0.01; *P < 0.05

Protein isoprenylation mediated by FTase plays an important role in the activation and function of small G proteins (especially the Ras superfamily). Lipid modification of the valence link by farnesylation enhances the hydrophobicity of Ras protein and promotes the localization of Ras from the cytoplasm to the inner surface of the cell membrane. This process is crucial for the further activation of Ras and related downstream signalling pathways.^32^ Among Ras GTPases, H‐Ras is only farnesylated by FTase, whereas N‐Ras and K‐Ras can be farnesylated or geranylgeranylated.^33^ Salirasib, farnesylthiosalicylic acid, competes with Ras for binding to Ras‐escort proteins and selectively disrupts the association of Ras proteins with the plasma membrane. In order to investigate whether FNTB overexpression affects the activity of Ras protein in cardiomyocytes and whether the change in Ras activity is due to the increase in farnesylation modification caused by FNTB, we added Ras inhibitor salirasib and control solvent separately after inducing cardiomyocytes to overexpress FNTB. Then, we detected the H‐Ras subcellular localization and Ras‐GTP activation in each group of cardiomyocytes after intervention. We investigated the membrane localization of active H‐Ras in cardiomyocytes with immunofluorescence analysis. As shown in confocal images (Figure 2A), strong plasma membrane staining of H‐Ras was observed in AdFNTB‐infected cardiomyocytes. Moreover, the subcellular distribution of H‐Ras induced after FNTB overexpression was profoundly blocked by salirasib treatment. Active Ras‐GTP levels were detected by G‐LISA assay. The Ras activation was significantly higher in the cardiomyocytes infected with AdFNTB than in those infected with AdGFP (Figure 2B). Furthermore, the results of G‐LISA assay also indicated that salirasib (the inhibitor of Ras) inhibited the FNTB overexpression‐induced activation of Ras. Thus, these results confirmed overexpression of FNTB in cardiomyocytes can lead to an increase in the farnesylation modification of the downstream small G protein Ras and an increase in active Ras‐GTP levels.

### 3.3 FNTB overexpression induces cardiomyocyte hypertrophy

**FIGURE 3:**
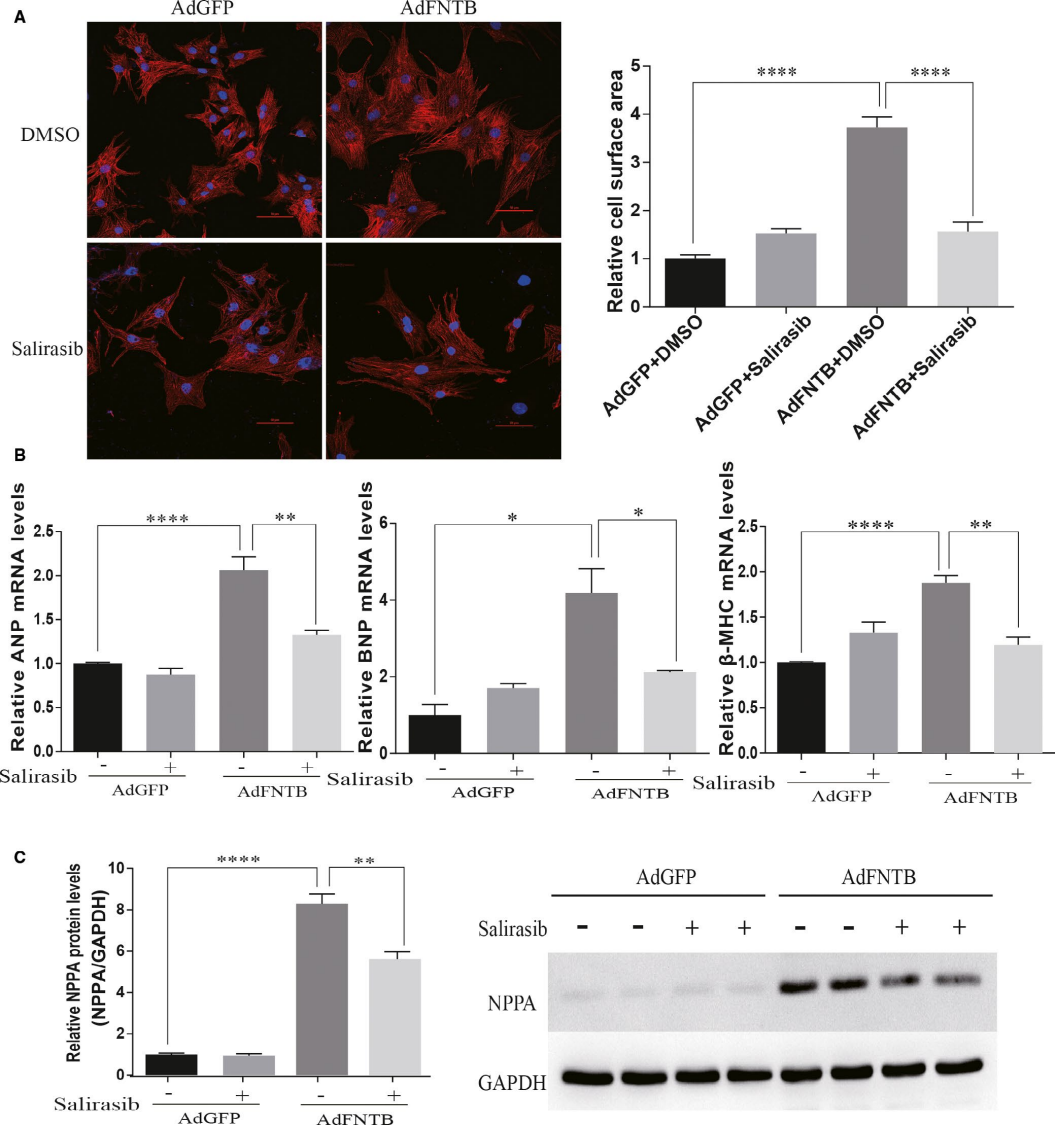
Cardiomyocyte hypertrophy induced by FNTB overexpression. A, The representative α‐actinin (red) immunofluorescence image of cultured neonatal cardiomyocytes infected with AdGFP or AdFNTB in the absence or presence of salirasib. DAPI (blue) was used as a nuclear counterstain. Scale bar: 50 μm. The cell surface area was evaluated by measuring a total of 100 cardiomyocytes derived from 3 independent experiments by using the ImageJ software. B, Gene expression levels of *ANP, BNP and β‐MHC* were detected by real‐time PCR. C, The protein level of NPPA was detected by Western blot analysis. ****P < 0.0001, **P < 0.01, *P < 0.05

To elucidate the role of FTase in cardiomyocyte hypertrophy, we imaged neonatal cardiomyocytes immunostained with α‐actinin and measured their cell surface areas. Overexpression of FNTB significantly increased the cardiomyocyte surface area (Figure 3A). The cardiomyocytes overexpressing FNTB showed hypertrophy along with an increase in the mRNA levels of atrial natriuretic peptide (*ANP*), brain natriuretic peptide *(BNP*) and β‐myosin heavy chain (*β‐MHC*), as evident from the results of RT‐qPCR (Figure 3B). *NPPA* protein expression was significantly increased following FNTB overexpression (Figure 3C). Furthermore, we evaluated whether the inhibition of Ras activation may in turn alleviate the development of cardiomyocyte hypertrophy induced by FNTB overexpression. The stimulation with salirasib (25 μmol/L) for 24 hours after 48 hours of transfection with adenovirus almost completely abrogated the cardiomyocyte hypertrophy induced by FNTB overexpression (Figure 3A). Up‐regulation in the mRNA levels of *ANP*, *BNP* and *β‐MHC* and the protein level of *NPPA* in response to FNTB overexpression was remarkably suppressed after salirasib treatment (Figure 3B,C). Taken together, these results suggest that the overexpression of FNTB and the up‐regulation of Ras activation in sequence play a vital role in cardiomyocyte hypertrophy.

### 3.4 Overexpression of FNTB inhibits cardiomyocyte viability

**FIGURE 4:**
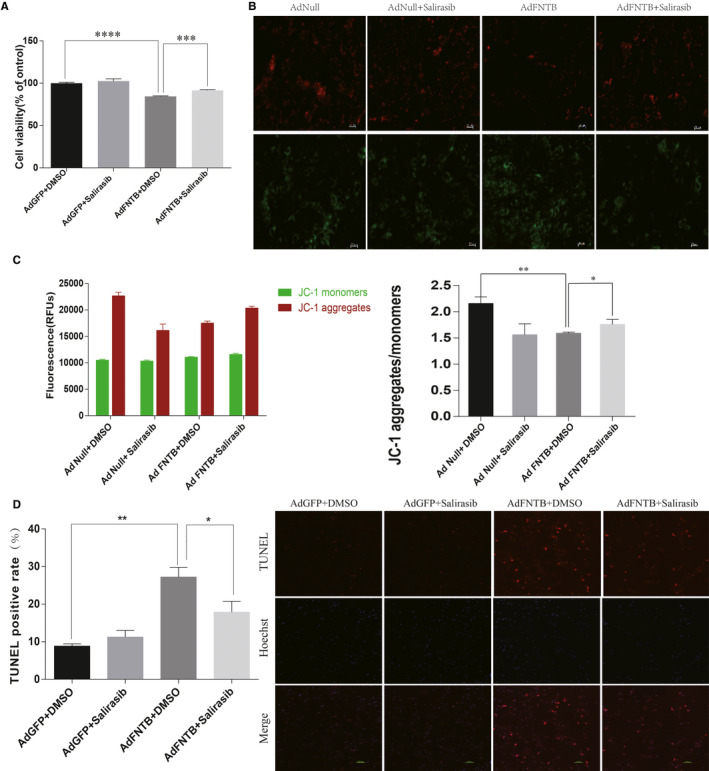
Overexpression of FNTB mediates cell survival, mitochondrial membrane potential ΔΨm and apoptosis in cardiomyocytes. A, Cell viability was determined with CCK‐8 assay. Mean ± SEM from 3 independent experiments. B, Mitochondrial membrane potential, evaluated with JC‐1. Red fluorescence indicates mitochondria in which membrane potential is maintained, whereas green fluorescence indicates depolarized mitochondria. Scale bar: 20μm. C, Mitochondrial membrane potential of purified mitochondrial detected by JC‐1. Fluorescence (RFUs) of JC‐1 monomer and aggregate was measured by fluorescence microplate reader. For the JC‐1 monomeric dye, λexcitation maximum is 514 nm and λemission maximum is 529 nm; for the J‐aggregate form of JC‐1, λexcitation maximum is ~485‐585 nm and λemission maximum is ~590 nm. D, TUNEL assay for apoptosis detection. Apoptotic cells were detected by staining with TF3‐dUTP (red). Nucleus was stained with Hoechst (blue). Scale bar: 100μm. The percentage of apoptotic cells was calculated from the ratio of TUNEL‐positive nucleus to the total cell nucleus stained with Hoechst (n ≥ 3). ****P < 0.0001,***P < 0.001, **P < 0.01

To determine the effect of FNTB overexpression on the viability of NRCs, the CCK‐8 cell viability assay was performed. As a result (Figure 4A), we found that FNTB overexpression significantly decreased the viability of cardiomyocytes as compared with the control AdGFP‐infected cells. Treatment of FNTB‐overexpressing neonatal cardiomyocytes with salirasib prevented the FNTB‐induced decrease in cell viability.

### 3.5 FNTB overexpression stimulates apoptosis in cardiomyocytes

The decrease in mitochondrial membrane potential ΔΨm is a landmark event in the early stage of apoptosis. The results in Figure 4B showed that the red fluorescence of the JC‐1 polymer in cardiomyocytes in the FNTB overexpression group is significantly weaker than the control group. The purified mitochondrial JC‐1 test results (Figure 4C) also confirmed that overexpression of FNTB can lead to a decrease in mitochondrial membrane potential ΔΨm of myocardial cells, while Ras inhibitor salirasib can alleviate this change. Transduction with AdFNTB significantly increased the number of terminal deoxynucleotidyl transferase dUTP nick end labelling (TUNEL)‐positive cardiomyocytes (Figure 4D).

**FIGURE 5:**
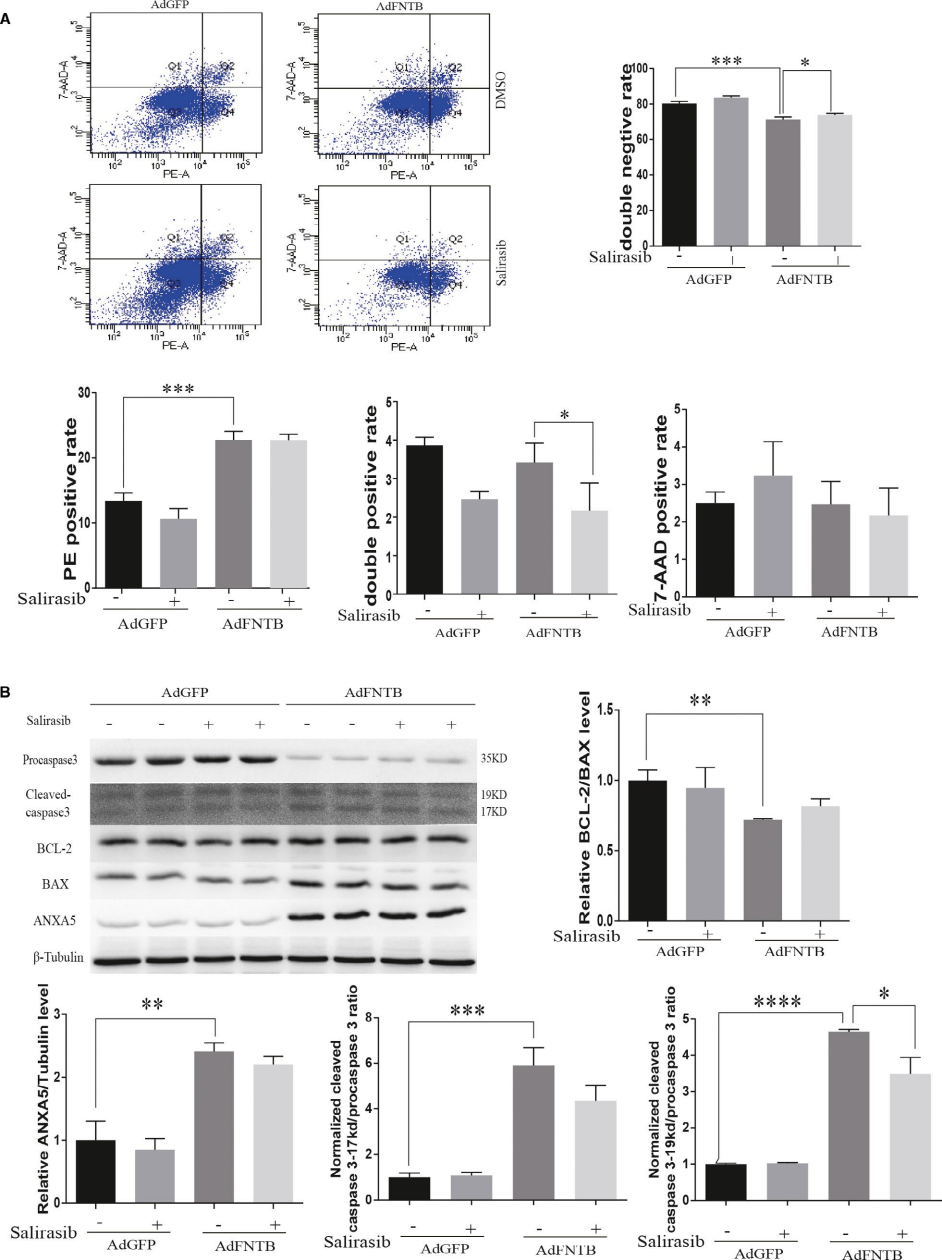
Overexpression of FNTB mediates cell survival. A, The apoptotic ratio of cardiomyocytes was measured with flow cytometry using ANXA5‐PE and 7‐AAD staining. B, ANXA5 and anti‐apoptotic Bcl‐2/pro‐apoptotic Bax, caspase 3 proteins were detected by Western blotting. ***P < 0.001, **P < 0.01, *P < 0.05

Consistent with these results, the population of viable cells determined with ANXA5‐PE and 7‐AAD‐negative staining was notably lower in FNTB‐overexpressing cardiomyocytes than in the AdGFP‐infected control group (Figure 5A). A significant increase in the population of cells undergoing early apoptosis, as indicated with ANXA5‐PE‐positive staining, was observed in AdFNTB‐infected cells as compared with the control group. The early apoptosis rate of myocardial cells in the FNTB overexpression group and the GFP control group was 22.8% and 13.4%, respectively. There was a statistically significant difference between the two groups. Western blot analysis for apoptosis showed similar findings, as evident from the increase in the expression of ANXA5 and pro‐apoptotic Bax/anti‐apoptotic Bcl‐2 proteins in the FNTB‐overexpressing cardiomyocytes (Figure 5B). Overexpression of FNTB also triggered the cleavage of procaspase‐3 into its p19 and p17 forms of the large subunit. Consistent with the results of TUNEL assay and Western blot analysis, flow cytometric analysis revealed the factual protective effect of salirasib against apoptosis with the increase in the number of viable cells by the double‐negative staining and a decrease in the number of end‐stage apoptotic and dead cells in ANXA5‐PE and 7‐AAD‐positive staining.

### 3.6 Overexpression of FNTB mediates autophagy in cardiomyocytes

**FIGURE 6:**
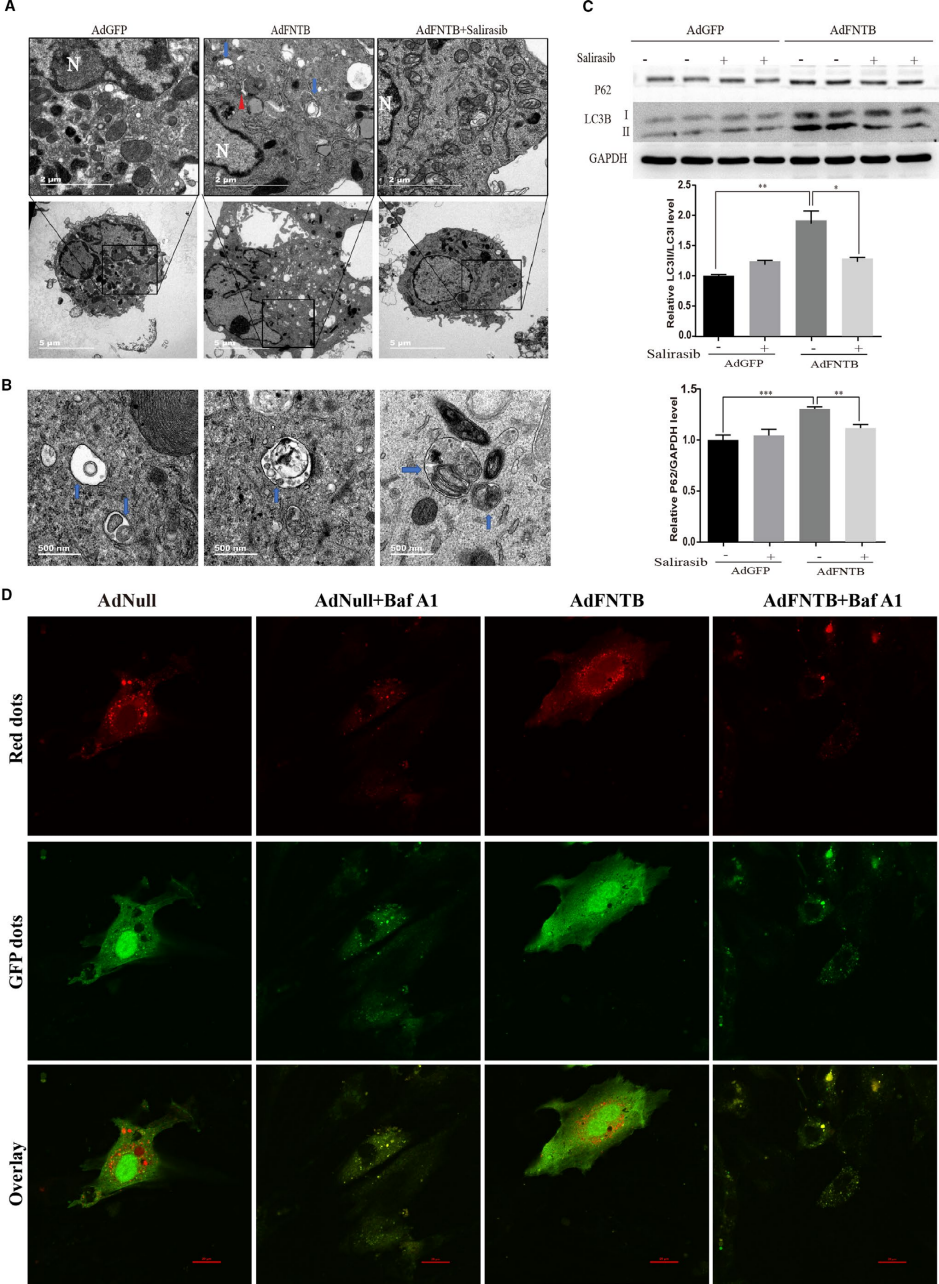
Overexpression of FNTB mediates the autophagy in cardiomyocytes. A, Representative transmission electron microscopy (TEM) images of neonatal cardiomyocytes. Arrows highlight autophagolysosomes (blue arrows). The red triangle indicates an early autophagic vesicle. N means nucleus. The scale bar in the original image represents 5 μm, and the scale bar in the enlarged image represents 2 μm. B, The amplified autophagosome structure observed in the overexpression group. The scale bar: 500 nm. Most of the autophagosomes are late autophagolysosomes. Some autophagosomes showed encapsulated undegraded mitochondrial structures and residual cell components. C, Western blot analysis of LC3B (upper band for LC3BI and lower band for LC3BII) and p62 expression. ***P < 0.001, **P < 0.01, *P < 0.05. D, Cardiomyocytes were cotransduced with tandem mCherry‐GFP‐LC3 and AdNull or AdFNTB for 96 h. Some cardiomyocytes were incubated with bafilomycin A1 (100 nmol/L) for 8 h. Red puncta indicate autolysosomes, and red and green (yellow) puncta indicate autophagosomes. Scale bar, 20 μm

JC‐1 test results showed that overexpression of FNTB caused a decrease in mitochondrial membrane potential of myocardiomyocytes, reflecting changes in mitochondrial membrane permeability and mitochondrial dysfunction. We performed TEM to confirm whether FNTB overexpression could interfere with the mitochondria and other ultrastructures in cardiomyocytes. Compared with the GFP control group, we found that the number of normal mitochondria in the cardiomyocytes of the FNTB overexpression group was reduced, the mitochondria structure was abnormal, and obvious autophagosomes were visible. Some autophagosomes showed encapsulated undegraded mitochondrial structures and residual cell components. TEM analysis also showed that the autophagic structures were evidently attenuated in FNTB‐overexpressing cells after the exposure to salirasib (Figure 6A). When we further amplified the autophagosome structure observed in the overexpression group, we can find that most of them are late autophagolysosomes (Figure 6B). Autophagy was further assessed by Western blot analysis for the expression of key proteins involved in autophagy, including LC3BII and p62. Levels of LC3 lipidation (LC3BII/LC3BI ratios) and autophagy substrate p62 significantly increased in FNTB‐overexpressing neonatal cardiomyocytes. Salirasib can reduce LC3BII/I ratio and p62 level induced by FNTB overexpression (Figure 6C).

There are two possibilities for the accumulation of autophagosomes and the increase in the level of autophagy‐related protein LC3II: increased autophagy activity or blocked autophagy flux. Autophagy flux was monitored by the use of tandem mCherry‐GFP LC3 fluorescence analysis. According to the number of red and yellow fluorescent puncta observed by confocal fluorescence microscope, autophagosomes and autophagolysosomes can be distinguished, reflecting the process of autophagy fusion and degradation, and assessing the state of autophagy flux. Figure 6D shows that the autophagolysosomes as red puncta in cardiomyocytes of the overexpressed FNTB group were significantly increased compared with the control group. After treatment with the late autophagy flux inhibitor bafilomycin A1 (100 nmol/L) for 8 hours, autolysosome formation as red puncta was significantly reduced, the fusion and degradation process of autophagosomes and lysosomes were blocked, and the autophagy flux that originally occurred in cardiomyocytes of the FNTB overexpression group was interrupted. These results suggest that FNTB overexpression in neonatal cardiomyocytes enhanced autophagy flux, and inhibitor of Ras alleviated the boosted autophagy level induced by overexpression of FNTB.

### 3.7 Effect of overexpressing FNTB on p38 MAPK and JNK signalling pathway

**FIGURE 7:**
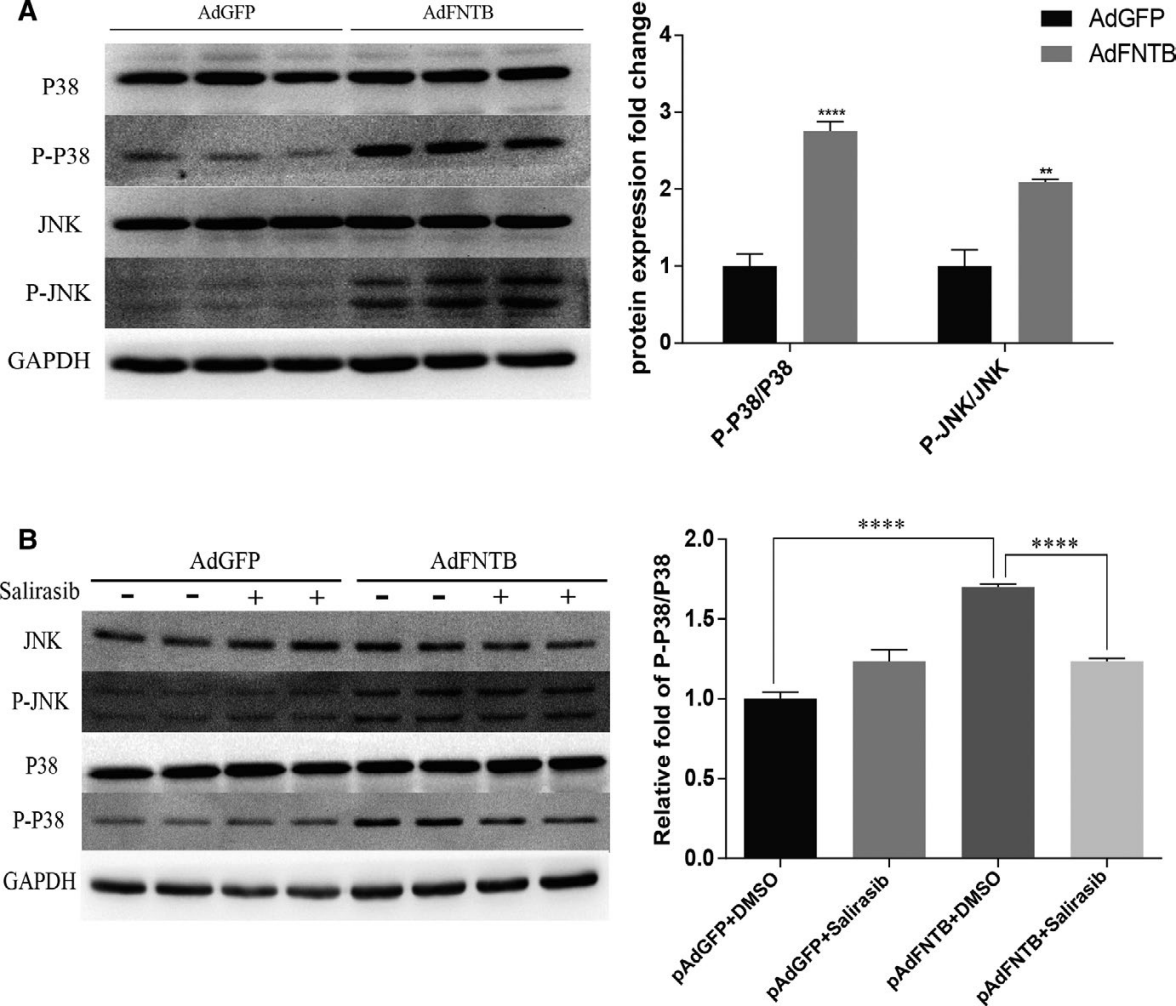
Effect of FNTB overexpression on the MAPK pathways. A, Representative immunoblots and statistics of c‐Jun N‐terminal kinase (JNK), p38, and total JNK, p38. Overexpression of FNTB in rat neonatal cardiomyocytes significantly up‐regulated the relative phosphorylation levels of JNK and p38. B, Salirasib significantly attenuated the p38 activation induced by FNTB overexpression. However, no significant reversal effect was observed on the phosphorylation of JNK. n ≥ 4, **** *P* < 0.0001, ** *P* < 0.01

Farnesyl modification catalysed by FTase enhance protein hydrophobicity and plasma membrane association, in which Ras cycles from an inactive Ras‐GDP to an active Ras‐GTP. Ras‐GTP then activates downstream related signalling pathways, including the MAPK pathway and kinase kinase kinase MEKK‐JNK pathway.^34^ Results of Western blot analysis showed that the phosphorylation levels of p38 MAPK and JNK in the overexpressed FNTB group were significantly increased (Figure 7A). The phosphorylation level of p38 in cardiomyocytes in the overexpression group with salirasib intervention was significantly lower than that in the overexpression group alone, but there was no statistically significant difference in the phosphorylation level of JNK between the two groups (Figure 7B).

## 4 DISCUSSION

In the current study, we used recombinant adenovirus overexpressing FNTB to infect primary cardiomyocytes of neonatal rats to explore the potential role and mechanism of FTase and the isoprenylation levels of its downstream small G protein Ras in myocardial remodelling. The results show that FNTB overexpression causes hypertrophy and promotes cardiomyocyte apoptosis and autophagic death, consistent with the activation of Ras and MAPK signal pathway. Ras inhibitor salirasib antagonizes farnesyl modification of Ras by competition, selectively disrupts the association of Ras proteins with the plasma membrane, inhibits Ras activation, improves cardiomyocyte hypertrophy induced by FNTB overexpression and increases the survival rate of cardiomyocytes in the FNTB overexpression group. Our data provide a new evidence that cardiomyocyte‐specific overexpression of FNTB could contribute to cardiac hypertrophy and death of cardiomyocytes in myocardial remodelling.

The physiological importance of the MVA pathway in cardiovascular diseases has been previously explained. As isoprenoid intermediates, farnesyl pyrophosphate (FPP) and geranylgeranyl pyrophosphate (GGPP) play a critical role in lipid attachment for the post‐translational modification of heterotrimeric small G proteins.^35^, ^36^ As another key enzyme in the MVA pathway, FTase mainly catalyses the farnesyl modification of downstream small G proteins (mainly Ras) and is closely related to the subcellular translocation and activation of Ras.^37^ Evidence has also suggested that the overexpression of the oncogenic Ras in cardiac myocytes could elicit a hypertrophic response, while the treatment with FTase inhibitor or the blockade of Ras expression could attenuate cardiac myocyte hypertrophy.^21^, ^38^ Previous studies of our team have shown that the tissue of the heart in the suprarenal abdominal aortic coarctation (AAC) group showed overexpression of farnesyltransferase‐beta (FNTB) and the activation of the downstream protein Ras was enhanced. FTase inhibitor (FTI) can alleviate myocardial fibrosis and partly improve cardiac remodelling in spontaneously hypertensive rats.^22^, ^24^ Together, these findings indicate the important role of FTase in cardiac remodelling. However, the exact role and mechanism of FTase in myocardial hypertrophy and remodelling are not fully understood. Herein, we provide a new evidence that the cardiomyocyte‐specific overexpression of FNTB induces cardiac hypertrophy in cultured primary rat neonatal cardiomyocytes, characterized with an increase in the size of cells and the levels of hypertrophy biomarkers *ANP*, *BNP* and *β‐MHC* at the mRNA level and NPPA at the protein level. In line with the previous studies, our data demonstrate that the inhibition of the overactivation of Ras could significantly attenuate the hypertrophic phenotype of cardiomyocytes induced by FNTB overexpression.^39^

In response to an insult, the population of myocytes decreases via various mechanisms during cardiac remodelling, including apoptosis, oncosis and autophagic cell death.^40^ Numerous studies have demonstrated the increase in the apoptosis of cardiocytes in the ventricular myocardium during the ventricular remodelling process and cultured hypertrophic cardiomyocytes.^41^, ^42^ Contrary to the role of apoptosis, autophagy was initially thought to alleviate the progression of contractile dysfunction and remodelling during hemodynamic overload and maintain cardiac homeostasis and function in most cases.^11^ Suppression of cardiac autophagy below the physiological level in the heart is associated with the progression of heart failure.^43^ Other investigators have also confirmed that hypertrophic response and cardiac dysfunction could be attenuated by increasing autophagy and reducing apoptosis during cardiac hypertrophy and remodelling.^44^ However, several studies in recent years have revealed that excessive autophagy contributes to pathological consequences in the cardiovascular system.^45^, ^46^ The results of the TUNEL assay and flow cytometric analysis of our study also showed the increase in the level of apoptosis in AdFNTB‐infected cardiomyocytes along with cardiomyocyte hypertrophy. ANXA5 protein has been shown to be up‐regulated during apoptosis activation; excessive level of myocardial ANXA5 could contribute to systolic dysfunction.^47^ The Western blot analysis of ANXA5 supported the results that the overexpression of FNTB induced apoptosis and injury in cardiomyocytes. The Bcl‐2 family members are grouped into the anti‐apoptotic Bcl‐2‐like proteins and pro‐apoptotic Bax‐like members that play an important role in apoptosis. In particular, Bcl‐2 proteins are implicated in the regulation of the crosstalk between apoptosis and autophagy.^48^, ^49^ The decrease in mitochondrial membrane potential ΔΨm by JC‐1 detection not only indicates the early apoptosis of cardiomyocytes, but also reflects the changes in mitochondrial membrane permeability and the mitochondrial dysfunction in cardiomyocytes of the FNTB overexpression group. TEM was performed to assess the mitochondria and other ultrastructures in cardiomyocytes. Accumulation of autophagosomes and mitochondrial autophagy can be observed in cardiomyocytes of the FNTB overexpression group under electron microscope. In order to clarify whether an increased autophagic process or blocked autophagy flux downstream was responsible for the accumulation of autophagosomes and LC3II level, the tandem mCherry‐GFP LC3 fluorescence was used to monitor autophagic flux. Red fluorescent dots representing autophagolysosomes increased and accumulated in cardiomyocytes, indicating that functional autophagy flux was activated after overexpression of FNTB. In the presence of lysosomal degradation inhibitor bafilomycin A1, the fusion degradation process between autophagosomes and lysosomes was blocked, and the red dots representing autophagosomes in cardiomyocytes in both the control group and the overexpressed group were significantly reduced. All these evidence indicate that after overexpression of FNTB, the level of autophagy flux in cardiac myocytes is enhanced; our data also demonstrate that the salirasib can alleviate autophagy abnormalities caused by FNTB overexpression.

Intracellular autophagy and apoptosis are not independent and unrelated regulatory processes. More and more studies have begun to focus on the ‘crosstalk’ between autophagy and apoptosis and the complex influence of the interaction on the occurrence and development of cardiovascular diseases.^50^ However, evidence about the two responses and their interplay is not fully defined and understood. In the process of myocardial remodelling, due to hypertrophy of myocardial cells, and extensive intracellular metabolic remodelling, mitochondria can be damaged due to toxic effects such as imbalance of energy metabolism, oxidative stress and calcium overload. Mitochondrial autophagy, as a kind of selective autophagy reaction against mitochondria, can clear the damaged mitochondria in the cell and participate in maintaining energy homeostasis and cell vitality, but excessive activation of autophagy or autophagy flux defects accelerates cell death.^51^ In our study, it was found that after FNTB overexpression, the mitochondrial membrane potential of cardiomyocytes decreased, suggesting that mitochondrial membrane permeability is abnormal. We also found that mitochondrial pathway endogenous apoptosis increased, and mitochondrial autophagy was observed under transmission electron microscope. In our study, the molecular machinery and ‘crosstalk’ between FNTB overexpression‐induced apoptosis and autophagy were not clarified due to our current inevitable restrictions in research.

The MAPK signalling cascade is classically initiated by the activation of small G proteins in cardiomyocytes, followed by the activation of successively acting protein kinases.^52^ At the cellular level, JNK and p38 kinases are generally considered as specialized transducers of stress or injury responses, including apoptosis and autophagy, while ERK1/2 kinase is more specialized for cell proliferation and differentiation.^53^, ^54^ Our result confirmed that the phosphorylation levels of p38 and JNK greatly increased in the AdFNTB‐infected group as compared with those in the AdGFP control group. Salirasib can suppress the up‐regulated phosphorylation level of P38 MAPK, but it has no significant effect on the phosphorylation level of JNK. Numerous evidence indicates that the increased phosphorylation level of MAPK pathway signalling is related to ventricular remodelling, and inhibition of MAPK pathway protein phosphorylation can relieve ventricular hypertrophy and improve heart function.^55^, ^56^ The roles of p38 and JNK MAPK in the control of the balance between autophagy and apoptosis have been identified as a strategy worth exploring for cell death. However, this view is still controversial, and more relevant research evidence is needed to supplement.^57^ As we realize the limitations of this study, we make a few assumptions and self‐inspection. (a) As one of the substrates activated by farnesylation, Ras may be not the only substrate activated upon FNTB overexpression. According to previous studies, complex interactions are involved in the activation of small GTPases such as Ras and non‐Ras.^58^, ^59^ Here, we assessed only the farnesyl modification and activation of Ras induced by overexpression of FNTB and could not focus on the activation or inhibitory effects of non‐Ras targets such as Rho. (b) Although primary neonatal cardiomyocytes are widely used for investigating molecular mechanisms and cellular processes involved in cardiac hypertrophy, our future work will involve gene editing animal models, which are more ideal research tools. This may help us further evaluate the effect of FTase in the pathological process of cardiac remodelling and heart failure. (c) Further research more concentrated on the molecular machinery and mechanisms of crosstalk between autophagy and apoptosis should be carried out.

In summary, our results indicate that overexpression of FNTB and its mediated protein isoprenylation of Ras induce cardiomyocyte hypertrophy and reduce the survival rate of cardiomyocytes by regulating apoptosis and autophagy. Antagonizing the level of Ras farnesylation modification can improve cardiomyocyte hypertrophy and increase the survival rate of cardiomyocytes. The underlying mechanism may be related to the Ras‐MAPK signalling pathway. These results provide a novel insight into the role of FTase as a potential target for cardiac hypertrophy, cardiac dysfunction and heart failure.

## CONFLICT OF INTEREST

The authors declare no conflict of interest.

## AUTHOR CONTRIBUTION

**Jie Ding:** Conceptualization (lead); Data curation (lead); Formal analysis (lead); Investigation (equal); Methodology (lead); Project administration (equal); Writing‐original draft (lead). **Yu Xiao Chen:** Data curation (equal); Formal analysis (equal); Investigation (equal); Software (equal); Visualization (equal). **Yan Chen:** Investigation (equal); Validation (equal); Writing‐review & editing (equal). **Yun Mou:** Formal analysis (equal); Investigation (equal). **Xiao Tong Sun:** Investigation (equal); Software (equal). **Dongpu Dai:** Methodology (equal). **Chenze Zhao:** Conceptualization (equal). **Jian Yang:** Conceptualization (equal). **Shenjiang Hu:** Funding acquisition (equal); Supervision (equal); Writing‐review & editing (equal). **Xiaogang Guo:** Funding acquisition (equal); Project administration (equal); Supervision (equal); Validation (equal); Writing‐review & editing (equal).

## Supporting information

Supplementary Material

## Data Availability

I confirm that my article contains a Data Availability Statement.
