# Supplementary material for: Overexpression of FNTB and the activation of Ras induce hypertrophy and promote apoptosis and autophagic cell death in cardiomyocytes
Source: J Cell Mol Med. 2020 Jun 24;24(16):8998–9011. doi: 10.1111/jcmm.15533 (PMC7417704; doi:10.1111/jcmm.15533)
Supplement: Supplementary file 1 — Supplementary Material [file JCMM-24-8998-s001.docx]

**Supplementary material**

**1.Isolation and culture of** **rat** **neonatal cardiomyocytes**

In brief, pups were sacrificed and the ventricles were excised and minced in cold D-Hanks (pH 7.4) solution. Enzymatic digestion was carried out with collagenase I (0.05% w/v, Sigma-Aldrich) and trypsin (ethylenediaminetetraacetic acid [EDTA]-free, 0.08% w/v, Invitrogen) in phosphate-buffered saline (PBS) at 37°C. The obtained cell suspension was filtered through a 75 μm strainer (Biologix) to remove any extracellular material and centrifuged at 1000 rpm for 5 min. Cells were pre-seeded in culture dishes with Dulbecco’s modified Eagle’s medium (DMEM, HyClone) containing 20% fetal calf serum (FBS, HyClone) and penicillin-streptomycin (100 U/mL penicillin and 100 mg/mL streptomycin) for 90 min to remove any non-myocytes. The unattached cardiomyocyte-enriched fraction was seeded in culture plates (Corning) coated with collagen type I (rat tail collagen, BD) at a density of 5 × 105 cells/cm2 and maintained in DMEM containing 20% FBS, 1% penicillin-streptomycin, and 0.1 mmol/L bromodeoxyuridine (Sigma-Aldrich) for 48 h. The culture medium was replaced with fresh DMEM containing 10% FBS and 1% penicillin-streptomycin. Cells were incubated at 37°C and 5% CO2.

**2. Generation of** **recombinant adenovirus and transfection**

The rat FNTB (NM_172034) CDS fragment was subcloned into the adenoviral shuttle vector pAdeno-MCMV between *Eco*RI and *Xba*I sites to construct a pAdeno-MCMV-FNTB recombinant transfer plasmid. The recombinant virus particles were obtained by co-transfecting HEK293 cells with the shuttle vector pAdeno-MCMV-FNTB and pAdMax™ genomic plasmid. The recombinant adenovirus particles were subsequently amplified in HEK293 cells and purified with cesium chloride density-gradient ultracentrifugation. The recombinant adenoviruses only containing GFP (AdGFP) were used as a negative control.

Neonatal rat cardiomyocytes (NRCs) were infected with recombinant adenovirus vectors as previously described[[1](#_ENREF_1),[2](#_ENREF_2)]. After 2 h of serum-free transfection, equal volumes of fresh complete medium were added to each well and NRCs were maintained in DMEM containing 5% FBS for further 20 h. The NRCs were exposed to dimethyl sulfoxide (DMSO) or salirasib (25 μM) for 24 h before experiment.

We infected cardiomyocytes with adenovirus vectors at MOIs ranging from 2.5 to 100 and examined the expression of GFP after 2 days with fluorescence microscopy（Figure 1B in main text）. The transfection efficiency was enhanced with an increase in MOI, and no green fluorescence was observed for the non-transfected control cells. The majority of cardiomyocytes expressed GFP upon exposure to the virus at MOI 30, as evident from the green fluorescence. No significant differences were observed between the groups infected at MOI of 30 and 100. No obvious cytotoxicity or cardiomyocyte death was detected after the application of AdGFP vector at an MOI of 100 within 96 h. Based on these results, we selected MOI 30 as the optimum dose for the infection of neonatal cardiomyocytes in the following experiments.

**3. Immunofluorescence staining**

For immunofluorescence experiments, NRCs were cultured on coverslips coated with collagen type I (rat tail collagen, BD) and then fixed with 4% paraformaldehyde (Solarbio, Beijing, China) for 25 min. The cells were permeabilized with 0.1% Triton X-100 in PBS for 20 min and blocked with 10% goat serum in PBS for 1 h to reduce any unspecific antibody binding. The cells were incubated with primary antibodies against sarcomeric alpha-actinin (mouse monoclonal, Abcam, 1:200, #ab9465), Troponin T-C(CT3) (mouse monoclonal, Santa Cruz, 1:50, #sc-20025), or H-Ras-specific antibody (rabbit polyclonal, Proteintech, 1:200, #18295-1-AP) in 1% bovine serum albumin (BSA) in PBST in a humidified chamber overnight at 4°C, followed by incubation with Alexa Fluor 594- or 488-conjugated secondary antibodies (goat anti-mouse or goat anti-rabbit, Invitrogen, 1:500) for 1 h at room temperature in the dark. Coverslips were mounted with a mounting medium fortified with 4′,6-diamidino-2-phenylindole dihydrochloride (DAPI, Abcam) and images were acquired with confocal scanning microscopy (Nikon A1R, Japan), followed by analysis with NIS-Elements Viewer software (version 4.20, Nikon, Japan). Quantitative analysis of images was carried out using ImageJ analysis software.

**4. Origins of antibodies for western blot analysis**

Primary antibodies against FNTB (Abcam, #ab109748), glyceraldehyde 3-phosphate dehydrogenase (GAPDH; Abcam, ab181602), natriuretic peptide A (NPPA; Abgent, # AM8592b), Bax(Proteintech group, #50599-2-Ig), Bcl-2(Proteintech group, #26593-1-AP), LC3B (Proteintech group, #18725-1-AP), p38(Proteintech group, #14064-1-AP), p62(Sangon biotech, #D222791), ANXA5 (Sangon biotech, # D220066), Caspase 3 (Cell Signaling, #9662), c-Jun N-terminal kinase (JNK1/2/3(T178); Bioworld, #BS3630), phosphorylated (p)-p38(Cell Signaling, #4511), p-JNK(Cell Signaling, #4668P).

**5.Western blot analysis**

After treatment, total proteins were immediately extracted from NRCs using a cell lysis buffer (Cytoskeleton) containing protease and phosphatase inhibitor cocktail (Roche). The lysate protein concentrations were measured with the BCA Protein Assay kit (Thermo Fisher) and equalized using ice-cold lysis buffer. Equal amounts of denatured proteins were separated with sodium dodecyl sulfate polyacrylamide gel electrophoresis (SDS-PAGE) and the separated bands were blotted onto polyvinylidene difluoride (PVDF) membranes (Millipore). Nonspecific protein binding was blocked using Tris-buffered saline with 0.1% Tween-20 (TBST) containing 5% nonfat milk for 1 h. The membranes were incubated with primary antibodies overnight at 4°C, followed by incubation with horseradish peroxidase-coupled secondary antibodies at room temperature for 1 h. Protein bands were visualized using enhanced chemiluminescence (ECL) detection kit (Millipore). Images were captured using Chemi XR 5 detection system (Syngene) and analyzed with ImageJ analysis software.

**6. Supplemental Tables**

| **Table 1** | | |
| --- | --- | --- |
| Primer sequences for RT-PCR. | | |
| Oligo Name | Forward(5' to 3') | Reverse(5' to 3') |
| FNTB | AGCCAAGCCACAGAAGGT | CGAAGGCACTGCTGAATG |
| β-MHC | TGGAAATGCGAAGACTGT | ATAGGGATGTAGCCGTTG |
| ANP | TGAGCCGAGACAGCAAACA | TCCAGGTGGTCTAGCAGGTT |
| BNP | AGTCTCCAGAACAATCCACGATGC | CCGGAAGGCGCTGTCTTGAG |
| GAPDH | AAGAAGGTGGTGAAGCAGGC | TCCACCACCCTGTTGCTGTA |

1. **Sun TT, He J, Liang Q, Ren LL, Yan TT, Yu TC, Tang JY, Bao YJ, Hu Y, Lin Y, Sun D, Chen YX, Hong J, Chen H, Zou W, Fang JY.** LncRNA GClnc1 Promotes Gastric Carcinogenesis and May Act as a Modular Scaffold of WDR5 and KAT2A Complexes to Specify the Histone Modification Pattern. *Cancer discovery*. 2016; 6: 784-801.

2. **Lu XY, Chen L, Cai XL, Yang HT.** Overexpression of heat shock protein 27 protects against ischaemia/reperfusion-induced cardiac dysfunction via stabilization of troponin I and T. *Cardiovascular research*. 2008; 79: 500-8.
